# Detection of *Helicobacter Pylori* infection by invasive and non-invasive techniques in patients with gastrointestinal diseases from Iraq: A validation study

**DOI:** 10.1371/journal.pone.0256393

**Published:** 2021-08-23

**Authors:** Rawaa A. Hussein, Mushtak T. S. Al-Ouqaili, Yasin H. Majeed

**Affiliations:** 1 Department of Clinical Laboratory Sciences, College of Pharmacy, University of Anbar, Al-Anbar Governorate, Ramadi, Iraq; 2 Department of Microbiology, College of Medicine, University of Anbar, Al-Anbar Governorate, Ramadi, Iraq; 3 Department of Internal Medicine, College of Medicine, University of Anbar, Al-Anbar Governorate, Ramadi, Iraq; Consiglio Nazionale delle Ricerche, ITALY

## Abstract

There is still no agreement on the gold standard technique for diagnosing of *H*. *Pylori* in Iraq, as well as a paucity of data on the validity of different diagnostic techniques. This study aimed to investigate the prevalence of this bacterium with different methods and compare them to the quantitative polymerase chain reaction (qPCR) as a golden standard technique among Iraqi patients. In total, 115 Iraqi patients strongly suspected of *H*. *pylori* infection were enrolled in the current study. Invasive techniques including rapid urease testing (RUT) and gastric tissue culture in addition to non-invasive techniques including ^14^C-Urea breath test (^14^C-UBT), stool antigen test (SAT), CagA-IgG serology, and qPCR were performed to confirm the *H*. *pylori* infection. The qPCR was used as the gold standard to estimate the sensitivity, specificity, positive and negative predictive values for the studied diagnostic tests. Overall, the prevalence rate of *H*. *pylori* in Iraqi patients was ranged from 47.8 to 70.4% using different methods. The positive results for each test were as follows: qPCR 81, (70.4%) UBT 79 (68.7%), SAT 77 (67%), RUT 76 (66.1%), Cag-IgG 61 (53%), and culture 55 (47.8%). The ^14^C-UBT showed the highest overall performance with 97.5% sensitivity, 97% specificity, and total accuracy of 97.3% followed by SAT, RUT, Cag-IgG, and culture method. Based on the accuracy of the studied methods for *H*. *pylori* detection, they can be arranged in order as follows: qPCR > UBT > SAT > RUT> CagA IgG > culture. Since a universal gold standard assay for the diagnosis of *H*. *pylori* has not yet been established in Iraq, the UBT may be recommended as first choice due to its higher performance compared to other methods.

## Introduction

*Helicobacter pylori* is a human pathogen that it’s infection is strongly related to many gastroduodenal diseases including peptic ulcer, atrophic gastritis, chronic active gastritis, mucosa-associated lymphoid tissue lymphoma in addition to non-cardiac gastric cancer [1]. The prevalence of *H*. *pylori* infection is 30%-50% in developed countries and 70%-90% in developing countries [2]. According to few studies in Iraq, accurate statistics and information on the prevalence of this bacterium are not yet available. Scattered studies in Iraq indicated a prevalence of 11.3 to 71.3% for *H*. *pylori* [3].

Many techniques are used to diagnose *H*. *pylori* infection which are divided into invasive and non-invasive ones. Invasive techniques include rapid urease test (RUT), microbiological culture, and biopsy based polymerase chain reaction [4]. It is well documented that non-invasive techniques include stool antigen tests (SAT), urea breath test (UBT), and serological investigations [5]. The factors which impact on the selection of the required technique strategy include sensitivity, specificity, clinical status, and cost-based issues. Therefore, depending on the clinical circumstances and patients history each test has its limitations, advantages, and disadvantages [4,6]. Non-invasive methods are favored in certain situations. These assessments are appealing due to their easiness and the potential to provide the results in a physician’s office within a few minutes of administration.

In the study of *H*. *pylori* infection, non-invasive methods are required to detect its incidence, transmission, and clearance [7]. The UBT is regarded as a gold standard non-invasive method for *H*. *pylori* diagnosis with higher sensitivity, specificity and accuracy in comparison with others. However, the specificity of UBT is decreased in the presence of other urease producing microorganism in the human gastrointestinal tract [4]. In comparison, the SAT method is the other main non-invasive technique with 94% and 97% sensitivity and specificity respectively [8]. Stool antigen tests lack accuracy because they are influenced by several factors, like antibiotics, proton pump inhibitors (PPIs), N-acetyl cysteine, bowel movements, and upper gastrointestinal bleeding [4,8]. Further, virulence factors have also been present to follow the prognosis of *H*. *pylori* infections, such as the presence of serum CagA, VacA, and GroEL antibodies in patients [9]. However, these serology factors cannot be used to differentiate between asymptomatic colonization status and active infection and either it is past or current *H*. *pylori* infection, and cannot be used to check the success of eradication of the bacterium [4,10].

Endoscopic biopsy, an invasive method for diagnosis of *H*. *pylori* infection, is recommended to be used in patients with dyspeptic symptoms. Endoscopy also provides an accurate and transparent picture of the gastric mucosa, although it does not provide clearer outcomes than most other tests [4,11]. RUT is another invasive test that used as the most routine technique in clinical practice. It is so important to know that for the high sensitivity to be obtained, it is necessary the bacterial load to be at least 10^5^ bacteria [12]. Thus, it is not recommended to be used in the follow up of post-eradication since this bacterial amount may not be found until about 30 days after therapy failure.

Another invasive technique is the culture. The microbiological culture from endoscopy based gastric biopsies, is consider a definite proof for *H*. *pylori* investigation. The culture of *H*. *pylori* from gastric biopsy is characterized by a high specificity with low sensitivity. The culture has 100% specificity, but with significant variation regarding sensitivity ranging from 85% and 95%. Due to the fastidious and delicate nature of this bacterium, i*n vitro* culture technique requires a certain transport media, specific incubation conditions, and growth medium for the routine diagnosis of *H*. *pylori* [8,10]. It is well recognized that the advantage of isolating *H*. *pylori* in the cultures under aseptic condition is predisposing for antibiotic susceptibility test of high quality to help clinician in the selection the most suitable antimicrobial agents to be used in the treatment [10].

Endoscopic based quantitative polymerase chain reaction (qPCR) has a superiority in the detection of low bacterial loads in addition to identification of clarithromycin resistance encoding genes due to specific mutations associated with such type of antimicrobial resistance [13]. It is well reported that PCR is characterized by high sensitivity and specificity (greater than 95%) in comparison with other conventional techniques. It is more accurate in detection of *H*. *pylori* in bleeding patients. Furthermore, PCR has the crucial role in the determination of point mutations encoding for resistance to *H pylori* in addition to the virulence factors like CagA and VacA [14].

There is still no agreement on the gold standard technique for diagnosing of *H*. *Pylori* in Iraq, as well as a paucity of data on the validity of different diagnostic techniques. Also, the epidemiological information on the prevalence of *H*. *pylori* is scarce in Iraq. Hence, this study aimed to investigate the prevalence of this bacterium with different methods and compare them to the qPCR as a golden standard technique among Iraqi patients.

## Materials and methods

### Ethics statement

All study techniques that involved patients were approved by the Ethical Approval Committee, University of Anbar, Ramadi, Iraq (approval number 122, November 20, 2019). Informed written consent was provided by all patients or their parents participating in the study.

### Specimen collection

In total, 115 patients with gastrointestinal diseases symptoms who referred to Ramadi Teaching Hospitals and Private Clinics in Iraq for routine upper gastrointestinal endoscopy, were enrolled in the current study from January 2020 until February 2021. Exclusion criteria were applied to patients who have received H2-receptor blockers, antimicrobial therapy, PPI and/or non-steroid anti-inflammatory drugs one month pre-endoscopy. Subjects with the following clinical conditions were also excluded from the study: cirrhosis, nephropathy in critical stages, and pregnancy. Information about demographic and socio-economic factors, and personal medical history of enrolled patients was previously collected by a questionnaire. Non-invasive test (SAT, UBT, and Cag-IgG) were evaluated in all patients. The gastric biopsy samples were obtained from the antrum and corpus of the stomach during routine endoscopy by an expert clinician (gastroenterologist). All biopsies were placed in sterile tubes containing brain heart infusion (BHI) broth medium (Oxoid, UK) and 5% of fetal bovine serum for transportation. The gastric biopsies were used for culture, RUT, and qPCR. The faecal samples in addition to sera from the study patients were handled immediately and kept at -20°C until used.

### Definition of *H*. *pylori* status

A positive result of qPCR was defined as a positive *H*. *pylori* infection. A negative *H*. *pylori* infection was confirmed when all tests performed gave concordant negative results.

### Rapid urease test (RUT)

The RUT was achieved by suspending 24.01 g of urea agar base in 950 ml of deionized water. After autoclaving and sterilization by Millipore filter paper, 50 ml of 40% urea solution was added. The constituents mixed well and poured into the tube laying at an angle. A biopsy was placed into a medium at 25 ^o^C. The change of color from the original yellowish color to a red through 24 hours is reflecting the positivity of the test [15].

### Bacterial culturing

Biopsies samples were cultivated on BHI agar (Oxoid, UK), consisting of 5% fecal bovine serum (Capricorn, South America), 7% horse blood, and antibiotics including nystatin, nalidixic acid, and vancomycin. Next, the plates were incubated at a temperature of 37°C under microaerophilic conditions with saturated humidity for 5–7 days, leading to a decrease of 0.1 oxygen within 2.5 hours and an increase of 7–15% CO_2_ within 24 hours (Anaerocult A; Darmstadt, Germany) [16]. All stock cultures were stored in BHI broth supplemented with 15% glycerol and 10% fecal bovine serum at -20°C. These preparations were thawed and sub-cultured for the subsequent experiments.

### ^14^C-Urea breath test (UBT)

Patients swallowed a test capsule that contains urea tagged with radioactive carbon 14 with water, or on an empty stomach, or two hours after eating. The UBT was carried out within 7 days of the endoscopy, before antibiotic treatment was given. The ^14^C labeled urea was detected by using HUBT-20P *Helicobacter pylori* detector (Headway, China). If the patient is infected with *H*. *pylori*, the test urea will be broken down and isotope-labelled carbon dioxide will be produced and is exhaled, which is then detected by the instrument [17].

### Stool antigen test (SAT)

This test was carried out by immunochromatographic assay, using *H*. *pylori* Ag Rapid Test CE (CTK Biotech, USA). It was performed for the qualitative detection of *H*. *pylori* antigen in the human fecal specimen, which indicates an active *H*. *pylori* infection. The procedure followed the manufacturer’s instructions [17]. A random stool specimen was collected in a clean, dry receptacle. The stool collection device was shake vigorously ensure a homogenous liquid suspension. The specimen and test components was brought to room temperature if refrigerated or frozen. The stool collection device was hold vertically and the cap was twist off. Two drops (70–90 μl) was dispensed of the solution into the sample well of the cassette and the solution was not overload. Results can be read at 10 minutes. The results will considered negative if only the C line developed and the test indicated no detectable *H*. *pylori* antigen in the specimen. Positive result appeared when the both C and T lines developed.

### Detection of CagA- IgG

The serum derived from a 5 ml of blood sample of each patients. The CagA test was done on these sera using Human *Helicobacter Pylori* Cytotoxin-Associated Gene A Protein IgG (HP-CagA-IgG) enzyme-linked immunosorbent assay (ELISA) Kit (Cusabio, USA) using the ELISA system (Human, Germany) according to the manufacturer’s instructions. The serum or plasma samples was diluted with sample diluent (1:1000) before test. A total of 100 μl of negative control, positive control, and diluted samples was added in each well of ELISA microplate. The microplate was covered by adhesive tapes and incubated for 30 minutes at 37°C. Each well was aspirated and washed three times. Then, 100 μl of HRP-conjugate (1×) was added to each well. The microplate was covered with the adhesive strip and incubated for 30 minutes at 37°C. The aspiration/wash process was repeated for five times as in step 6, 90 μl of TMB substrate was added to each well and incubated for 20 minutes at 37°C and protected from light. Finally, 50μl of stop solution was added to each well, the plate was gently tap to ensure thorough mixing. The optical density of each well was determined within 10 minutes, using a microplate reader set to 450 nm. The results were classed as positive if CagA antibody (IgG) concentrations were > 0.9 U/ml.

### Molecular technique

#### Extraction of DNA from biopsy specimens

DNA was extracted using SaMag ^TM^ Tissue DNA extraction kit, and SaMag-12^TM^ automatic nucleic acid extraction system was used for the extraction of genomic DNA (Samaga, Cepheid, Italy). The Quantus^TM^ Fluorometer (Promega, USA) was used to measure the concentration of extracted nucleic acid to detect the quality of the sample for further applications.

#### Quantitative real-time PCR

This was achieved using the thermal cycler (Sacace—Italy) with a qPCR kit for the qualitative detection of *H pylori* (Sacace—Italy). PCR program is consisting of one cycle of 15 minutes at 95°C, followed by 45 cycles of 10 seconds at 95°C, 30 seconds at 60°C, and a final cycle of 10 seconds at 72°C.

### Data analysis

All data were analyzed using the SPSS^TM^ software, version 22.0 (IBM Corporation, Armonk, NY, USA). Sensitivity and specificity parameters, positive and negative predictive values, the likelihood ratio (LR): the odds (likelihood) of being infected if the test result was positive (LR+) and the odds of being infected if the test result is negative (LR-) were calculated.

## Results

The patients included 80 males (69.6%) and 35 females (30.4%) with an age range from 18–70 years. Of the total number of patients, 53 (46.1%) had antral gastritis, 2 (1.7%) had combined gastritis and duodenitis, 7 (6.1%) had duodenitis, 1 (0.9%) had gastric tumor or adenocarcinoma, 4 (3.5%) had hiatus hernia, 19 (16.5%) had combined gastric ulcers and duodenal ulcers, 1 (0.9%) had esophagitis, and 28 (24.3%) patients had dyspepsia (Table 1). The distribution of people with gastrointestinal disorders by age is listed in Table 2. Most patients were in the age range of ≤ 37 years.

**Table 1:** The number of studied patients with gastrointestinal diseases symptoms subjected to endoscopy based on gender.

	Antral gastritis	Combined gastritis and duodenitis	Duodenitis	Gastric tumor, Adenocarcinoma	Hiatus hernia	Combined gastric and duodenal ulcers	Esophagitis	Dyspepsia
**Total: Female/Male (n)** **Total: Female/Male (%)**	53: 14/39 46.1%: 12.2/33.9%	2: 1/1 1.7%: 0.86/0.86	7:2/5 6.1%: 1.7%/4.4%	1: 1/0 0.9%	4: 1/3 3.5%:0.86%/2.6%	19: 6/13 16.5%:5.2%/11.3%	1: 0/1 0.9%	28: 10/18 24.3%: 8.7%/15.6
Age–Year(Mean ± SD)	34.62 ± 13.290	57.00 ± 16.971	28.71 ± 13.086	66.00	40.25 ± 10.178	36.58 ± 16.249	20.00	39.61±14.441

**Table 2:** The distribution of people with gastrointestinal disorders by age.

Age groups) year)			≤ 37	38–53	54 ≤	Total
Disease	Antral gastritis	Count	35	11	7	53
		% within Disease	66.0%	20.8%	13.2%	46.1%
	Combined antral gastritis and duodenitis	Count	2	0	0	2
		% within Disease	100.0%	0.0%	0.0%	1.7%
	Combined gastric and duodenal ulcers	Count	13	3	3	19
		% within Disease	68.4%	15.8%	15.8%	16.5%
	Duodenitis	Count	5	1	1	7
		% within Disease	71.4%	14.3%	14.3%	6.1%
	Esophagitis	Count	1	0	0	1
		% within Disease	100.0%	0.0%	0.0%	0.9%
	Gastric tumor, adenocarcinoma	Count	0	0	1	1
		% within Disease	0.0%	0.0%	100.0%	0.9%
	Hiatus hernia	Count	2	2	0	4
		% within Disease	50.0%	50.0%	0.0%	3.5%
	Dyspepsia	Count	13	5	10	28
		% within Disease	46.4%	17.9%	35.7%	24.3%
Total		Count	71	22	22	115
		% within Disease	61.7%	19.1%	19.1%	100.0%

The results of each diagnostic test are shown in Table 3. Out of 115 biopsy specimens, 81 (70.4%) were positive by qPCR and 34 (29.6%) were negative. The positive results for other tests were as follows: UBT 79 (68.7%), SAT 77 (67%), RUT 76 (66.1%), Cag-IgG 61 (53%), and culture 55 (47.8%). As it is clear from the results, the most reliable test in comparison with qPCR was the UBT with the lowest false negative and false positive cases. Also, the culture method with the highest false positive and false negative cases was considered the most inefficient test.

**Table 3:** The results of each *Helicobacter pylori* diagnostic test compared to qPCR.

Technique	*Helicobacter pylori* diagnosis n (%)				
	Positive	Negative	False positive	False negative	Total
**qPCR** *	81(70.4%)	34 (29.6%)	0 (0.0%)	0 (0.0%)	115 (100%)
**UBT** *	79 (68.7%)	33 (28.7%)	1 (0.9%)	2 (1.7%)	
**SAT** *	77 (67%)	31 (27%)	3 (2.6%)	4 (3.4%)	
**RUT** *	76 (66.1%)	32 (27.9%)	2 (1.7%)	5 (4.3%)	
**Serology CagA- IgG**	61 (53%)	29 (25.3%)	5 (4.3%)	20 (17.4%)	
**Culture**	55 (47.8%)	27 (23.5%)	7 (6.1%)	26 (22.6%)	

*RUT, Rapid urease Test; UBT, Urea breath test; SAT, Stool antigen test; qPCR, quantitative real-time polymerase chain reaction.

The Table 4 shows the sensitivity, specificity, positive and negative predictive values, and the likelihood ratio for each test. In comparison with qPCR, the UBT showed the highest overall performance with 97.5% sensitivity, 97% specificity, and total accuracy of 97.3% followed by SAT, RUT, Cag-IgG, and culture method. The culture method with 67.9% sensitivity, 79.4% specificity, and total accuracy of 71.3% showed the lowest performance.

**Table 4:** Test performance for each *Helicobacter pylori* diagnostic test compared to qPCR.

Test	Sensitivity (%) (95% CI)	Specificity (%) (95% CI)	Positive predictive value (%) (95% CI)	Negative predictive value (%) (95% CI)	Positive likelihood ratio (%) (95% CI)	Negative likelihood ratio (%) (95% CI)	Test accuracy (%) (95% CI)	Disease prevalence (%) (95% CI)
**UBT** *	**97.5 (91.4–100)**	**97.0 (84.7–99.9)**	**98.8 (92–99.8)**	**94.3 (80.7–98.5)**	**33.2 (4.8–228.8)**	**0.03 (0.006–0.1)**	**97.4 (92.6–99.5)**	**70.4 (61.2–78.6)**
**SAT** *	**95.0 (87.8–98.6)**	**91.2 (76.3–98.1%)**	**96.3 (89.7–98.7)**	**88.6 (74.8–95.3)**	**10.8 (3.7–31.8)**	**0.05 (0.02–0.1)**	**93.9 (87.9–97.5)**	
**RUT** *	**93.8 (86.2–98)**	**94.1 (80.3–99.3)**	**97.4 (90.8–99.3)**	**86.5 (73.2–93.8)**	**16.0 (4.2–61.3)**	**0.07 (0.03–0.2)**	**93.9 (87.9–97.5)**	
**CagA- IgG**	**75.3 64.5–84.2**	**85.3 (68.9–95.0)**	**92.4 (84.3–96.5)**	**59.2 (49.2–68.5)**	**5.1 (2.3–11.6)**	**0.3 (0.2–0.4)**	**78.3 (69.6–85.4)**	
**Culture**	**67.9 (56.6–77.8)**	**79.4 (62.1% - 91.3%)**	**88.7 (80.0–93.9)**	**50.9 (42.0–59.8)**	**3.3 (1.7–6.5)**	**0.4 (0.3–0.6)**	**71.3 (62.1–79.4)**	

*RUT, Rapid urease Test; UBT, Urea breath test; SAT, Stool antigen test.

## Discussion

It is well recognized that the selection of diagnostic technique to investigate of *H*. *pylori* infection depends on the strains and prevalence of this bacterium in an endemic areas in addition to the advantages, disadvantages and accessibility for each technique [8]. In the clinical circumstances, a rapid and cost-effective determination technique for the surveillance of *H*. *pylori* is always required. So far, there is no comprehensive study to evaluate the prevalence of *H*. *pylori* infection in Iraqi patients through various methods. The current study was the first that investigated different invasive and non-invasive techniques for detection of *H*. *pylori* in Iraqi patients with gastrointestinal diseases symptoms.

It is well recognized that UBT is one of the major non-invasive diagnostic tools for the detection of active *H*. *pylori* infections. The test is used worldwide, is non-invasive and can be used in children and pregnant women, with millions of subjects screened every year [18]. The results of UBT showed that 68.7% (79/115) of studied patients had *H*. *pylori* that was almost similar to previous report by AL-Saad et al. [19] from Iraq who reported a prevalence rate of 67.1% for *H*. *pylori* infections with UBT method. However, our prevalence rate was much higher than report by Altamimi et al. [20] from Jordan using UBT method [14.6% (48/328)]. This discrepancy may be due the differences in studied population or the ages of participants. In order to evaluate the performance of this method, it is suggested that UBT be performed in various homogeneous age populations with a larger number of patients. In this study, a comparison of the sensitivity and specificity of UBT with qPCR, another diagnostic method has been carried out. The sensitivity and specificity were 97.5% and 97% respectively, which was very close to the rates observed by Atkinson et al. [6] and Rahim et al. [21]. The goal for the use of the ^14^C urea breath was to detect its sensitivity and specificity in diagnosing *H*. *pylori* compared to qPCR. Our results showed that the ^14^C urea breath test performed better than SAT, RUT, culture, and serology for diagnosis of *H*. *pylori*. In agreement with the current research, Alzoubi et al. [22] from Jordan compared three *H*. *pylori* diagnosis methods and concluded that the sensitivity, positive predictive /negative predictive values, and accuracy of UBT were higher than those for SAT compared to culture method. It is well known that false negative results can be due to low colonization of *H*. *pylori* in the stomach, which leads to low *H*. *pylori* urease levels, or to the patient’s taking PPIs and antibiotics before the examination [22]. Further, the presence of other urease-producing pathogens in the stomach also causes false positive results [8]. This test cannot provide information about genotypes and antibiotic resistance. Some medications including bismuth-containing compounds, antibiotics, and PPIs, could decrease the test sensitivity through reduction of the organism density or urease activity [10]. Thus, the study also depends on culture and on specific *H*. *pylori* diagnostic gene-based.

Regarding SAT method, a previous study has reported that this test is promising as preliminary diagnostic tool and it is useful for the follow up after treatment for the progression of *H*. *pylori* infection [23]. Using SAT test, the prevalence rate of 67% was revealed for *H*. *pylori* infection. This result was closely similar to the recent report by Galal et al. [24] from Egypt who stated 64.6% occurrence rate using SAT method. However, in contrast to this study, Al-Mashhadany et al. [25] reported much lower prevalence (11.3%) from Kurdistan region, Iraq using SAT method. These differences may be due to variation in socioeconomic status, educational level, nutritional habits, and hygiene states of studied regions. In this study, the stool antigen test revealed 95% and 91.2% sensitivity and specificity respectively at which they were close to the study results observed by Miftahussurur et al. [10]. In this test, false-negative findings similar to the UBT occur due to low bacterial load, and the use of PPIs, antibiotics, or bismuth [4]. However, the SAT method does not require fasting, and recently some variants of it have been commercially available that are not affected by PPIs. Also, various studies have shown the effectiveness of this method in diagnosing infected patients from treating patients, as well as its efficacy in evaluating the eradication of *H*. *pylori* infection [4,22]. Although this study showed that the SAT method was in the second place after UBT in term of sensitivity compared to qPCR, it is claimed, this test can be superior to UBT technique [4].

The serological detection of *H*. *pylori* infection with a CagA containing strain of *H*. *pylori* using anti-CagA ELISA is the non-invasive diagnostic technique for assessing the potentiality of the virulency and risky of the study strain. The reliability of CagA serology as a predictive test for determining the CagA genotype of the infecting strain is important because various serological assays are now available [25]. The serological part of this study reflected the decline of the specificity with 85.3% in comparison with others. That result is being very close to those observed by Atkinson et al. [6] and Amgalanbaatar et al. [26]. Anti-CagA antibodies are not always produced in the serum of people with *H*. *pylori*. Also, it has been hypothesized that various populations may have varied immune responses to *H*. *pylori* [27]. Cover et al. used ELISA to detect CagA-expressing *H*. *pylori* and correlated the serological results with detection of the CagA gene [28]. They found considerable discordance between ELISA and the molecular detection of CagA, especially for patients infected with CagA-negative *H*. *pylori* strains. They suggested that the interpretation may be due to mixed infection of unknown origin with CagA-positive and negative strains. It has been observed that ELISA kits differ in terms of sensitivity and specificity for the detection of *H*. *pylori* infection within a country. In each country or region, an ELISA kit with a domestic representative strain as an antigen is thought to be more appropriate [26]. In Iraq, all ELISA kits for *H*. *pylori* are imported and there is no domestic kit yet. In the current research, the false negative cases of anti-CagA were 17.4%. It is suggested that false negative ELISA results may have several possible explanations. First, the CagA gene is functional while mutations might have arisen in other loci within the cag PAI which modified the capability for the cell to export active CagA protein. This causes a lack of seropositivity. Second, the absence of anti-CagA antibodies in such patients may be due to sequence variation in CagA resulting in different epitopes [29]. It could also be due to the low level of antibodies produced against the antigen used in the ELISA kit [30]. Generally, the inability to differentiate between current and past infection may interpret the low accuracy of serological tests [4].

Another test that showed good efficacy compared to qPCR was the RUT method. Using RUT, the prevalence rate of 66.1% was stated for *H*. *Pylori*. This result was higher than a report by Aftab et al. [31] from Bangladesh (43.6%). In the present study, the sensitivity and specificity of the RUT were 93.8% and 94.1% respectively, which was so close to those identified by other authors [6,10,31]. The false negative was 4.3%. The recent use of PPIs and the existence of intestinal metaplasia are considering the most common causes for false negative results [29]. Approximately 10^5^ bacteria must be present in the biopsy sample for a positive result and anything that reduces the bacterial density, such as the use of antibiotics, bismuth-containing compounds, or PPIs may result in false-negative results [32]. Also, urease activity decreased in the coccoid form of *H*. *Pylori*, resulting in a negative urease test [10]. False positives are very rare and when present may be due to the presence of other urease-containing organisms such as *Proteus mirabilis*, *Citrobacter freundii*, *Klebsiella pneumoniae*, *Enterobacter cloacae*, and *Staphylococcus aureus* [32].

The current research revealed the sensitivity and specificity of 67.9% and 79.4% for culture method, respectively. However, Atkinson et al. [6] demonstrated the sensitivity and specificity of 60% and 100% for this method, respectively. Another study by Aftab et al. [31] reported the sensitivity and specificity of 92.1% and 100% for *H*. *pylori* culture, respectively. The sensitivity of the culture method to detect *H*. *pylori* in the adult population ranges from 62.7% to 96.3% in the studies performed [33]. Despite the fact that the culture method is considered a gold standard for diagnosis, it is difficult to utilize it alone as a routine diagnostic approach. *H*. *pylori* positivity can be found in cases of growth because of the low sensitivity of the culture technique. Also, the absence of growth does not rule out the possibility of *H*. *pylori* infection. Despite its long use, culture remains a challenge because of the fastidious nature of the bacterium with particular growth requirements regarding the environment and atmosphere [33]. Altering pH or the PPIs indirectly interferes with *H*. *pylori* distribution in the stomach. In our study, exclusion criteria were PPIs or H2-receptor antagonists and antibiotics 4 weeks before the beginning of the study, similarly to another study and recommendations [34]. The current results showed a rate of 22.6% for false negative cases of culture method. This may be due to the fact that *H*. *pylori* can exist in two forms, an actively dividing spiral form and a coccoid form. Coccoid forms have been described as “viable but non-culturable” [33].

Molecular techniques are superior to other methods in terms of test speed, no limitation on sample transport, and high accuracy. The PCR technique is being developed to investigate the presence of microorganism directly from the clinical samples. Compared with conventional PCR, real-time PCR has several advantages, such as short working time, high specificity and low risk of contamination [8,35]. Quantitative results from real-time PCR in our study showed that the sensitivity and specificity were 100% and 100% respectively. The qPCR result in this study was very close to those observed by Johannessen et al. [36]. Deng et al. reported that the sensitivity of real-time PCR was 100%. [34]. Also, in agrrement with the current study, Peng et al. [37] demonstrated the specificity of qPCR as 100% and sensitivity as 97.1%.

## Conclusion

Overall, the current study found that the prevalence rate of *H*. *pylori* in Iraqi patientswas ranged from 47.8 to 70.4% using different invasive and non-invasive detection methods. Based on the accuracy of the studied methods for *H*. *pylori* detection, they can be arranged in order as follows: qPCR > UBT > SAT > RUT> CagA IgG > culture. Since a universal gold standard assay for the diagnosis of *H*. *pylori* has not yet been established in Iraq, the UBT may be recommended as first choice due to its higher performance compared to other methods. Also, because most medical centers cannot afford expensive real-time equipment, the use of a fast and relatively inexpensive non-invasive method such as UBT can be used as a powerful primary screening tool. This study concluded markedly the role of real-time PCR as a more sensitive, reliable and accurate than another diagnostic study method. In spite of the better sensitivity for real-time PCR than for conventional PCR which make it more superior to be used, the statistical significance is still limited. Real-time PCR is faster and provide many observed advantages among conventional PCR. It allows detection of low bacterial loads as well as the identification of resistant genes and specific point mutations mediated clarithromycin, fluoroquinolones and other antimicrobial resistance [6]. Currently, the clinical use of PCR-based testing is reduced by some factors like high costs, but the high diagnostic performance in the pre-and post-treatment setting, with the additional option of identifying clarithromycin-resistant strains, render it an excellent recent and future diagnostic technique [6]. Due to increasing drug resistance of *H*. *pylori* to most common therapies, it is essential to set up a rapid and valid test for screening of antibiotic resistance genes in this bacterium [38]. The diagnosis of *H*. *pylori* should be carried out in light of the clinical setting. Also, a combination of assays is so necessary. When patients have an upper endoscopy with biopsy harvesting, PCR, especially real-time PCR, offers several advantages over culture. Real-time PCR is recommended in patients who have an upper endoscopy with biopsy in comparison with culture at which it is high accurate, reliable, time consuming and with high sensitivity. The study suggests that the gold standard technique for diagnosis of *H*. *pylori* is real time-PCR supported by precise culture under aseptic condition especially with patients with no response to antimicrobial chemotherapy. Also, it is required in antibiotic resistance surveillance and for identification and evaluation of effective *H*. *pylori* therapies.
